# The Role of Advanced Cardiovascular Imaging Modalities in Cardio-Oncology: From Early Detection to Unravelling Mechanisms of Cardiotoxicity

**DOI:** 10.3390/jcm12154945

**Published:** 2023-07-27

**Authors:** Isabella Leo, Mahesh Vidula, Giandomenico Bisaccia, Maria Cristina Procopio, Roberto Licordari, Maria Perotto, Giulia La Vecchia, Nikolaos Miaris, Paco E. Bravo, Chiara Bucciarelli-Ducci

**Affiliations:** 1 Royal Brompton and Harefield Hospitals, Guy’s and St Thomas’ NHS Foundation Trust, London SE1 7EH, UK; i.leo@rbht.nhs.uk (I.L.);; 2 Department of Experimental and Clinical Medicine, Magna Graecia University, 88100 Catanzaro, Italy; 3 Division of Cardiovascular Medicine, Department of Medicine, University of Pennsylvania, Philadelphia, PA 19104, USApaco.bravo@pennmedicine.upenn.edu (P.E.B.); 4 Divisions of Nuclear Medicine and Cardiothoracic Imaging, Department of Radiology, University of Pennsylvania, Philadelphia, PA 19104, USA; 5 Department of Neuroscience, Imaging and Clinical Sciences, “G. d’Annunzio” University of Chieti-Pescara, 66100 Chieti, Italy; 6 Department of Clinical and Experimental Medicine, University of Messina, 98122 Messina, Italy; 7 Department of Biomedical and Dental Sciences and of Morphological and Functional Images, University of Messina, 98122 Messina, Italy; 8 Department of Cardiovascular and Pulmonary Science, Catholic University of Sacred Heart, 00168 Rome, Italy; 9 School of Biomedical Engineering and Imaging Sciences, Faculty of Life Sciences and Medicine, King’s College London, London WC2R 2LS, UK

**Keywords:** cardiovascular magnetic resonance, nuclear imaging, cardioncology, cardiotoxicity

## Abstract

Advances in cancer therapies have led to a global improvement in patient survival rates. Nevertheless, the price to pay is a concomitant increase in cardiovascular (CV) morbidity and mortality in this population. Increased inflammation and disturbances of the immune system are shared by both cancer and CV diseases. Immunological effects of anti-cancer treatments occur with both conventional chemotherapy and, to a greater extent, with novel biological therapies such as immunotherapy. For these reasons, there is growing interest in the immune system and its potential role at the molecular level in determining cardiotoxicity. Early recognition of these detrimental effects could help in identifying patients at risk and improve their oncological management. Non-invasive imaging already plays a key role in evaluating baseline CV risk and in detecting even subclinical cardiac dysfunction during surveillance. The aim of this review is to highlight the role of advanced cardiovascular imaging techniques in the detection and management of cardiovascular complications related to cancer treatment.

## 1. Non-Invasive Imaging in Cardio-Oncology: General Considerations

**Figure 1:**
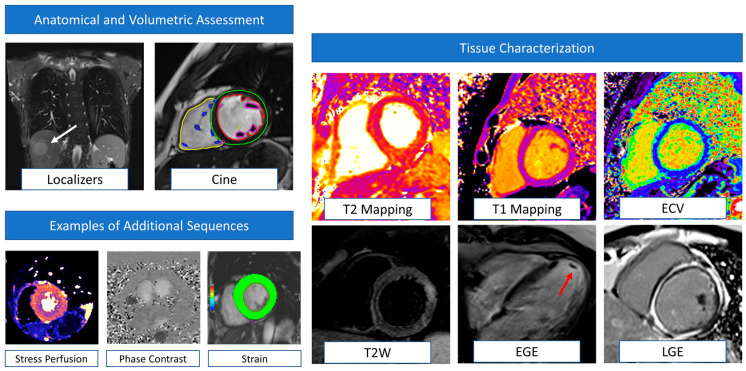
Examples of CMR sequences in cardio-oncology. Anatomical localizers and cine sequences represent the core of CMR evaluation, allowing the detection of extra-cardiac abnormalities (white arrows) and volumetric and function assessment (multi-colored regions of interest). This basic protocol is also very quick (~20–30 min) and useful when dealing with uncompliant or sick patients or in serial surveillance scans. CMR can also detect myocardial oedema (T2 weighted images) and fibrosis (T1 mapping, ECV, EGE, and LGE). Information derived by tissue characterization has been demonstrated to be helpful in enhancing the sensitivity of cardiotoxicity diagnosis. In addition, EGE sequences are particularly helpful in detecting intracardiac thrombi (red arrow). According to clinical suspicion, the CMR protocol could be tailored to the patient and include additional sequences such as stress perfusion imaging (to assess large and small vessel coronary disease), phase contrast (for flows and valvular function evaluation), and strain sequences. CMR: cardiovascular magnetic resonance. ECV = extracellular volume. EGE = early gadolinium enhancement. LGE = late gadolinium enhancement.

Several definitions of cardiotoxicity or cancer therapy-related cardiac dysfunction (CTRCD) have been provided over the years. Nevertheless, the unifying element for diagnosis is the detection of a new cardiac dysfunction defined as either reduction in the left ventricular ejection fraction (LVEF) or, more recently, in the global longitudinal strain (GLS) [1]. Both American and European guidelines highlight the role of the baseline imaging assessment to provide a pre-treatment assessment of LV systolic function with LVEF quantification [2]. This will establish not only a reference baseline to use in serial re-evaluations but will also allow a cardiovascular (CV) risk assessment with identification of any pre-existing cardiomyopathy. The assessment of diastolic parameters is also recommended in this population. In fact, although conflicting results exist in the literature, recent evidence demonstrated that de novo or worsening diastolic disfunction assessed by echocardiography are associated with a subsequent reduction in LVEF in patients treated with anthracyclines/trastuzumab [3]. Three-dimensional (3D) transthoracic echocardiography (TTE) should be the first-line imaging modality for LVEF assessment, with two-dimensional (2D) TTE used when 3D-TTE is not available or feasible [1]. However, the LVEF assessment should be carefully interpreted, taking into account loading conditions that can change during surveillance. In approximately 20% of patients fulfilling the CTRCD diagnosis, the reduction in LVEF was driven by a decrease in end-diastolic volumes associated with volume depletion [4]. Cardiovascular magnetic resonance (CMR) can provide an accurate assessment of LV systolic function and volumes when echocardiography is suboptimal or inconclusive [5]. Strain measurements by speckle tracking echocardiography are recommended at baseline and can predict cardiotoxicity early [6]. Similarly, myocardial strain by fast-strain encoded CMR (SENC) has demonstrated excellent reproducibility and lower variability compared to other CMR strain techniques, with accurate detection of early cardiac systolic dysfunction [7,8]. The unique advantage of CMR in this particular clinical contest is the possibility to provide myocardial tissue characterization, namely T2-weighted/T2 mapping sequences for the detection of myocardial oedema and T1 mapping, late gadolinium enhancement (LGE) sequences, and extracellular volume fraction (ECV) estimation for fibrosis detection. Despite CMR representing the gold standard for ECV quantification, a recent CT-derived ECV showed an excellent correlation with CMR-derived ECV values, providing a promising alternative for patients with CMR contraindications [9]. Examples of CMR sequences and their clinical application in cardio-oncology are summarized in Figure 1. 

Radionuclide techniques, particularly with positron emission tomography (PET) imaging, have also become key in pre-clinical and clinical studies of CTRCD. For example, myocardial perfusion imaging (MPI) with myocardial blood flow (MBF) quantification is crucial for the investigation of derangements of the coronary circulation that may result from cancer therapies, including radiation therapy. Moreover, cardiovascular molecular imaging has evolved significantly over the past few decades due to the advent of PET and radio-labeled imaging probes that can elucidate the biomolecular events that underlie clinical phenotypes; thus, providing new insights into the pathophysiology, diagnosis, management, and risk stratification of individuals with CTRCD.

## 2. Chemotherapy-Induced Cardiotoxicity

Anthracycline chemotherapy (AC) is currently prescribed for a wide range of malignancies and exhibits a well-known cumulative-dose cardiotoxicity [10,11]. The risk of heart failure after AC is 5% at a cumulative dose of 400–450 mg/m^2^ up to 48% at 700 mg/m^2^ [10,11]. The therapeutic mechanisms of action involve direct interference with DNA or RNA synthesis and transcription, inhibition of protein synthesis, mitochondrial damage, and an increase in reactive oxygen species (ROS) with subsequent additional damage to the DNA. Recently, it has also been postulated that anthracyclines could potentiate anti-tumor activity by triggering direct tumor cell death, enhance immune effector cell activation, and eliminate immunosuppressive myeloid-derived suppressor cells [12]. All these mechanisms are not specific to cancer cells and also occur in cardiomyocytes, particularly rich in mitochondria and susceptible to AC damage [11]. The cardiac injury could rarely manifest as acute toxic myocarditis with inflammatory infiltrates and myocardial oedema, increased risk of life-threatening arrhythmias, and signs and symptoms of acute heart failure [13]. Instead, vacuolization and fibrosis are the histopathological markers of chronic AC-induced damage [11]. The risk of heart failure remains high after months or years from the completion of treatment, with an overall mortality of 60% 2 years after diagnosis [14]. Advanced imaging techniques are particularly useful in detecting subclinical changes before the development of overt cardiac dysfunction, being of paramount importance during surveillance. 

### 2.1. Role of Cardiac Magnetic Resonance Imaging

A baseline assessment of LVEF and CV risk factors is mandatory to assess overall suitability to AC therapy, stratify patients according to their CV risk, and plan a tailored follow-up schedule [15]. As already mentioned, CMR can evaluate systolic function when other techniques are inconclusive or discordant [15]. However, during surveillance, LVEF impairment is usually a late marker of AC cardiotoxicity, occurring only when the myocardial damage is quite extended and all the compensative mechanisms to maintain an adequate systolic function are no longer sufficient [16]. CMR is particularly useful in this context due to its unique property of tissue characterization. Increased native T1, T2, and ECV have been observed after 6 weeks of treatment in a rat model treated with doxorubicin [17]. Another pre-clinical study conducted in AC-treated pigs identified T2 relaxation-time as the earliest biomarker of anthracycline-induced cardiotoxicity [18]. This parameter was significantly increased after only 6 weeks of treatment, when T1 mapping and ECV were still completely normal and changed only at a later stage, concurrently with the development of overt motion abnormalities [18]. The imaging data also pair with the pathology findings of intra-cardiomyocyte oedema and vacuolization in the early phase, without extracellular expansion. Therefore, T2 mapping seems to be the parameter able to detect intracellular changes before the development of interstitial fibrosis and cardiomyocyte loss. Of note, the observed vacuolization was reversible at this early stage with regression of vacuolization and normalization of T2-relaxation-time after AC interruption, emphasizing that early T2 prolongation occurs at a reversible disease stage [18]. These findings demonstrate the clinical potential of this CMR imaging biomarker for tailored anthracycline therapy [18]. Despite being interesting, these findings were not confirmed in a clinical study enrolling 30 sarcoma patients undergoing serial CMR evaluation at baseline, 48 h after the first AC dose and at completion of treatment [19]. In this study, T2 mapping values failed to predict cardiotoxicity, whereas significantly lower T1 values were observed in patients with subsequent cardiac dysfunction before and after AC exposure (1002.0 ± 37.9 vs. 956.5 ± 29.2 ms, *p* < 0.01) [19]. Although a pathophysiological explanation of these early changes was not clear, the authors raised the hypothesis that the decreased native T1 times reflected the increase in lipid intracellular content related to ROS production and lipid peroxidation [19]. Both T1 mapping and ECV have also been associated with adverse remodeling, reduced LV mass, and reduced exercise capacity during cardiopulmonary exercise testing in a cohort of survivor pediatric patients, even in the presence of normal LVEF [20]. A significant increase in relative contrast enhancement has been also observed only 3 days after AC administration and a >5-fold relative increase was able to predict significant LVEF reduction at 28 days [21]. After the acute phase, interstitial and/or replacement fibrosis seems to be the major determinant of LV dysfunction [22]. LGE can accurately identify myocardial fibrosis with a pattern that usually correlates with the underlying disease. However, overall, LGE seems to be a relatively uncommon finding in AC-treated patients, described in less than 1 in 10 patients with reduced LVEF [23,24]. More importantly, LGE had no association with major adverse cardiovascular events (MACE) at follow-up. A significantly prognostic association was instead found with reduced LV mass [23]. The right ventricle can also be involved in AC related cardiotoxicity with impaired RV function detected in approximately 1/3 cancer survivors [25]. A significant increase in RV ECV has been also observed after AC and correlates with a decline in RV mass-index [26]. However, tissue characterization remains challenging in daily clinical practice in the RV due to its very thin walls. Cardiomyocyte death and diffuse fibrosis eventually led to abnormal myocardial deformation that is accurately identified by a reduction in peak longitudinal strain and circumferential strain measured by CMR tagging with higher sensitivity when compared to STE echocardiography [27]. Circumferential strain by feature tracking also demonstrated an excellent correlation with subsequent LVEF decline observed at 3 months of follow-up in AC-treated patients (r = −0.49; *p* < 0.0001) [28]. Compared to tagging, feature tracking has the advantage of not requiring dedicated sequences and providing much faster semi-automated analysis [8]. 

Anti-human epidermal growth factor receptor 2 (HER2) is a monoclonal antibody used in HER2-positive breast cancer, causing cardiotoxicity in nearly 30% of patients, with an even greater risk when used in combination with AC [29,30]. Anti-HER2-related cardiotoxicity is usually reversible and mainly causes an impairment of contractility more than cell death [31]. A transient increase in inflammatory biomarkers, such as growth differentiation factor-15 (GDF-15) and matrix metallopeptidase-2 (MMP-2), has been observed, suggesting that reversible oedema and inflammation could also play a role in HER-2 related toxicity [32]. The reversibility of cardiac dysfunction, with the potential of full recovery after drug interruption, reinforces the importance of imaging surveillance. Myocardial oedema has been demonstrated by T2-weighted images early after Trastuzumab administration [32]. Subepicardial LGE has also been described by Fallah-Rad et al. in all patients experiencing Trastuzumab cardiotoxicity [33]. However, data coming from another cohort show LGE in only 10% of AC and/or trastuzumab patients with a wide range of patterns and localization, including ischemic distribution in a considerable proportion (64.5%). The same study also described LGE in cancer patients not undergoing AC or anti-HER2 therapy, suggesting that LGE might not be directly related to cardiotoxicity but may represent an incidental finding [34]. In addition, a decrease in absolute values of CMR strain has been reported in this context and predicts subsequent overt CTRCD [35,36].

### 2.2. Role of Radionuclide Imaging

Multigated acquisition (MUGA), or equilibrium radionuclide angiography (ERNA), was the initial radionuclide technique used to monitor LV function in patients receiving AC. However, this is now used less frequently than echocardiography or CMR, which do not require radiation exposure and can provide additional information regarding cardiac structure and function [37]. Current research focuses on the use of radionuclide molecular imaging techniques for the (early) detection of treatment-related cardiac injury (Table 1).

^18^F-fluorodeoxygluocose (FDG) is a glucose analogue that enters the cells through various types of glucose transporters (GLUT), and intracellular retention is dependent upon the enzymatic activity of hexose-6-phosphate-dehydrogenase within the endoplasmic reticulum [38,39,40]. FDG is taken up by metabolically active cells and is a sensitive molecular target for the investigation of cancer-related cardiotoxicity since metabolic derangement, tissue injury [41], inflammation [42], and hypoxia/ischemia [43] are potent stimuli for GLUT 1 and GLUT 3 expression. In one study, doxorubicin dose-dependently increased myocardial FDG uptake (after at least 6 h of fasting) in patients receiving treatment for Hodgkin lymphoma, particularly in the presence of low baseline cardiac FDG uptake, a finding that was also evident and reproducible in mice fed a standardized diet [44]. The same group also reported in a subsequent study an inverse association between myocardial FDG uptake and LVEF in patients receiving doxorubicin [45]. While the underlying mechanisms of myocardial glucose upregulation induced by doxorubicin are multifactorial and could not be fully elucidated, it is possible that, in part, they might be related to a metabolic switch from the known cytotoxic effects of doxorubicin to inhibit fatty acid oxidation and mitochondrial function [46], but also through the production of free radicals and excessive oxidative stress [47]. These findings raise the question of whether routine FDG-PET studies ordered for cancer surveillance could potentially also be used to monitor for cardiotoxicity [48]. However, this strategy seems unlikely as pharmacologic intervention (e.g., insulin administration) may be required prior to FDG administration in order to standardize glucose uptake in the heart. 

There are several other molecular imaging targets that have been evaluated in pre-clinical studies, which may help identify subclinical myocardial injury. ^99m^-Technetium (Tc)-annexin binds to phosphatidylserine, which is exposed in early apoptosis, and studies in doxorubicin-treated rats have shown increased myocardial uptake of this marker prior to the onset of ventricular dysfunction [49,50]. Increased expression of caspase 3, an enzyme involved in apoptosis, has also been identified using ^18^F-CP18 (a caspase 3 substrate) in rats treated with doxorubicin, suggesting that imaging of the apoptotic cascade may result in earlier detection of chemotherapy-related myocardial injury [51].

^123^I-metaiodobenzylguanidine (MIBG), a marker of myocardial sympathetic innervation, has been evaluated in patients treated with anthracyclines since damage to the sympathetic nervous system is hypothesized to contribute to the pathophysiology of patients with heart failure. Prior research has suggested that a reduction in ^123^I-MIBG myocardial uptake may precede impairment in left ventricular function, and pre-clinical data indicate that there may be an injury to sympathetic neurons in anthracycline-mediated cardiotoxicity [52,53]. Studies have compared the ratio of uptake between the heart and mediastinum and the washout rate [54]. While some studies found differences in these parameters between control subjects and anthracycline-treated patients [55,56], a more recent analysis did not show an association between ^123^I-MIBG myocardial uptake and changes in LVEF [57], suggesting that more work is required prior to using this imaging target in clinical practice. Other tracers under investigation for myocardial innervation imaging include ^18^F-flubrobenguane [58] and ^18^F-dihydroxyphenylalanine [59].

Direct cardiomyocyte injury and sarcolemmal damage can be imaged using antimyosin antibodies (^111^In-antimyosin), with prior studies demonstrating increased myocardial uptake with increasing doses of doxorubicin, preceding myocardial dysfunction and correlating with a reduction in LVEF [60,61,62]. However, this technique is limited by the commercial availability of this tracer [63]. The production of reactive oxygen species (ROS) and subsequent inflammation, injury, and myocyte apoptosis has also been proposed as a potential etiology of doxorubicin-mediated cardiotoxicity [64]. In a rodent model, superoxide production was detected using ^18^F-dihydroethidium (DHMT), and increased ROS production was noted to be an early marker of LV dysfunction. Similar findings were subsequently reported in a large animal model [65]. However, the translation of this molecular imaging target to humans remains to be proven.

Administration of taxane-based chemotherapy results in derangements in fatty acid metabolism due to impairment of microtubules [66]. ^123^I-15-(p-iodophenyl)-3-(R,S)-methylpentadecanoic acid (^123^I-BMIPP), a fatty acid analogue, has been studied in patients with lung cancer treated with taxanes, and reduced uptake was associated with a reduction in LV function, raising the possibility that this marker can be used to predict and detect cardiotoxicity [67]. Finally, preliminary studies have used ^68^Ga-fibroblast activation protein alpha inhibitor (^68^Ga-FAPI) to detect myocardial fibroblast activation in patients undergoing surveillance PET scans for cancer staging [68,69]. Additional work is required to understand how this tracer can be used to identify early myocardial fibrosis and chemotherapy-related cardiotoxicity.

## 3. Radiation-Induced Cardiotoxicity

Radiation therapy (RT) is the cornerstone of several haematological and solid malignancies. Despite several attempts over the last few years to reduce its potentially harmful effect, RT is still associated with an increased relative risk of cardiac mortality ranging from 2.2 to 12.7% and an almost five-fold increased risk of heart failure [70,71]. A clear dose–effect relationship has been described, with a linear increase in major coronary event rate by 7.4% per Gray of exposure (95% confidence interval, 2.9 to 14.5; *p* < 0.001) and no dose considered actually safe [72]. The CV adverse effects usually appear years or even decades after irradiation, with a higher risk in the case of left-sided RT, concomitant CV risk factors, or chemotherapy [73,74]. Radiation exposure induces endothelial damage with increased vascular permeability, the release of inflammatory markers, and adhesion molecules, leukocyte infiltration, and fibrin deposits [75]. The result is vessel obstruction, dysfunction and, therefore, ischemia, particularly at the microvascular level. The pro-inflammatory environment also stimulates collagen production through the transforming growth factor-β (TGF-β) pathway promoting myocardial fibrosis and cardiac dysfunction, with a further contribution to microvascular ischemia [75]. There is new evidence that RT could be regarded as a trigger of the systemic anti-tumor immune response, inducing a series of biological effects which are deemed to be systemic, immune-mediated, anti-tumor effects [76]. Ionizing radiation can enhance tumor antigen release and presentation, promoting activation of immune cells, increasing the density of tumor-infiltrating lymphocytes, facilitating recognition of tumor cells by T cells, and augmenting the anti-tumor effect, as well as leading to pro-inflammatory cytokine release [77]. Early recognition of these mechanisms could potentially help in identifying patients at risk that may benefit from closer surveillance. 

### 3.1. Role of Cardiac Magnetic Resonance Imaging

**Figure 2:**
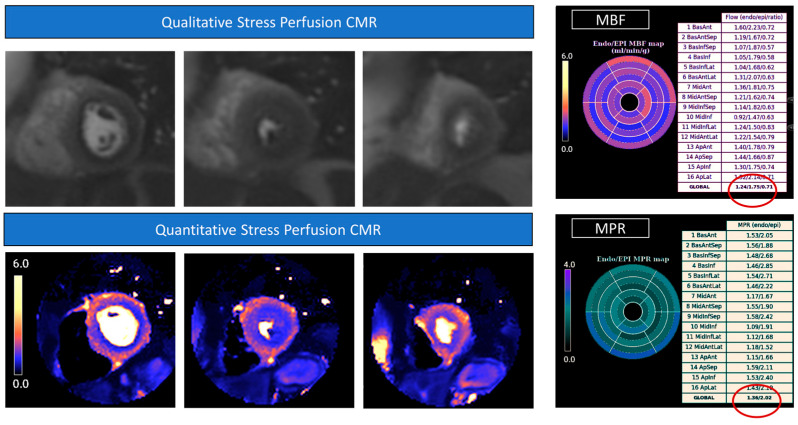
Stress perfusion Cardiovascular Magnetic Resonance. Raw perfusion images showed a diffuse hypointense «darker» area in all the coronary territories representing a myocardial perfusion defect during drug-induced (adenosine) hyperemia. The diffuse subendocardial perfusion defect is also highlighted in the colored perfusion maps, where hypoperfused areas are shown in blue. The quantitative analysis showed significantly reduced myocardial blood flow (MBF) values, particularly in the subendocardium (1.24 mL/min/g), and concomitant reduced subendocardial myocardial perfusion reserve (MPR) values (1.36).

All patients should undergo a thorough baseline assessment of CV risk factors and LVEF before RT, using echocardiography as a first-line imaging modality [1,15,78,79]. Imaging surveillance starts after 5 years in high-risk patients and instead of after 10 years in all the others [78]. However, recent studies have demonstrated changes in diastolic function parameters after 3 years, suggesting that an earlier evaluation may be considered [80]. Strain evaluation by CMR seems promising in this regard. In an animal model irradiated with 24 Gy, the myocardial strain was early impaired at 10 weeks, despite a normal systolic function [81]. Changes in circumferential strain were more substantial than longitudinal and radial strain, with the greatest reduction observed in the lateral wall reflecting the higher degree of vacuolization and necrosis found in this area compared to other myocardial segments [81]. Takagi et al. reported elevated native T1 and ECV values at 6 months in a group of 14 patients who underwent a combined chemotherapy–radiotherapy treatment (1183 msec ± 46 vs. 1257 msec ± 35; 26% ± 3 vs. 32% ±3; adjusted *p* < 0.01) [82]. However, another study conducted in a larger sample (*n* = 28) of RT-treated patients found no association between cardiac doses and native T1 values or LGE presence at a longer follow-up (mean 46.4 months). Therefore, the authors suggest that the early changes observed by Takagi’s group could have been influenced by concomitant chemotherapy [83]. Nevertheless, in another cohort a significant reduction in LV stroke volume index and septal non-ischemic LGE have been described in 78% patients after RT at 1.5 years of follow-up [82]. These data have been confirmed by Umezawa and colleagues, who described LGE in the irradiated myocardial segments of approximately half of the patients at a median follow-up of 23.5 months [84]. Interestingly, no LGE was found in the myocardial segments outside the irradiation field. Van der Velde et al. investigated CMR changes in a cohort of 80 long-term survivors of Hodgkin lymphoma. At a mean of 20 ± 8 years of follow-up, they found significantly reduced LVEF and longitudinal, radial, and circumferential strain using the feature tracking technique. In addition, native T1 values were also significantly higher compared to healthy controls, with LGE present in 11% of survivors [85]. The results of the ongoing CareBest trial will provide further insights into the prognostic role of these CMR parameters [86]. The pericardium is also often affected by irradiation either in the form of acute pericarditis or, chronically, as pericardial thickening and constriction. CMR is particularly useful in distinguishing between the two conditions, as LGE of the pericardium is usually associated with an active inflammatory process [87]. In addition, real-time free-breathing cine images allow the assessment of ventricular interdependence when constriction is suspected [88]. Valvular leaflets can also be damaged as a result of radiation exposure, with possible valvular dysfunction [74]. Despite its lower temporal resolution when compared with echocardiography, CMR can accurately assess valvular function by cine images and phase-contrast sequences. A two- to seven-fold higher risk of myocardial infarction and a four- to seven-fold higher risk of CAD has also been described in patients treated with RT [89]. Stress CMR could not only accurately assess scarring derived from established myocardial infarction but also identify underlying ischemia as hypointense “darker” areas after gadolinium contrast injection during pharmacological hyperaemia [90,91]. This functional test outperforms SPECT in both sensitivity and specificity (86.5/83.4% vs. 66.5/82.6%, respectively) and provides similar clinical outcomes when compared to invasive fractional flow reserve (FFR) in guiding revascularization [92,93]. Moreover, a novel respiratory motion-corrected myocardial perfusion technique with automated in-line perfusion mapping allows the quantification of myocardial blood flow during hyperaemia and at rest and the subsequent estimation of myocardial perfusion reserve. Quantitative perfusion mapping increases the overall sensitivity of perfusion CMR, particularly for the detection of microvascular ischemia, representing a promising tool for the evaluation of RT sequelae [94,95] (Figure 2). 

### 3.2. Role of Radionuclide Imaging

**Figure 3:**
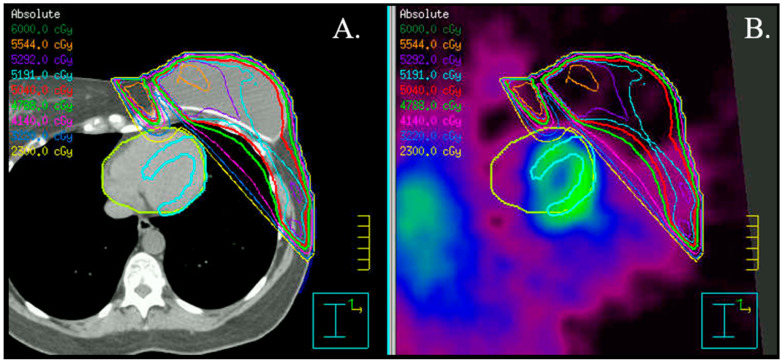
Radiation plan in a patient with left-sided breast cancer. Axial fused CT (**A**) and SPECT (**B**) images, including visualization of isodoses. Please notice the proximity of the left ventricular apical segments to the radiation field.

Myocardial perfusion imaging, particularly if coupled with MBF quantification, can potentially provide important insights into the integrity and function of the coronary circulation. In addition, it can also identify areas of interstitial and replacement fibrosis in the heart that can result in collateral damage from radiation therapy delivered to the thorax and breast tissue (Figure 3). 

**Figure 4:**
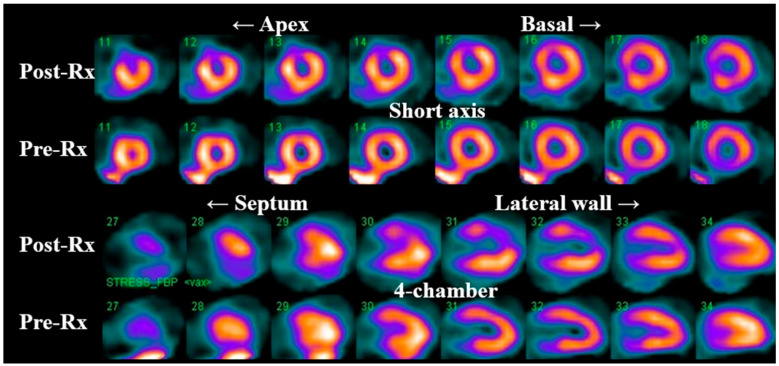
Myocardial SPECT images before and 6 months after radiation therapy (Rx). Please notice the development of a significant perfusion deficit in the apical, anterior wall.

Prior studies (Table 2) using single photon emission computerized tomography (SPECT) have observed a high incidence of new myocardial perfusion deficits (up to 60% in some series) as early as 6 months in patients receiving left breast/chest wall RT compared to pre-RT SPECT scans [96,97]. These perfusion deficits preferentially involve the anterior wall and apex (Figure 4) [97] and remain relatively stable at 12- and 18-month follow-up post-RT [97]. There is also a correlation between the development of perfusion deficits with the volume of irradiated heart [98] and the onset of cardiovascular symptoms [99]. 

From a mechanistic perspective, a recent mouse model of radiation-induced cardiotoxicity revealed that single radiation doses of 20, 40, or 60 Gy delivered to the LV apex resulted in perfusion SPECT deficits in the area of radiotherapy 8 weeks later [100], providing evidence for a significant vascular component to radiotherapy-induced cardiac injury, and corroborated histologically by coronary vessel thickening and perivascular fibrosis [100].

In agreement, focal apical LV FDG uptake was observed in the irradiated field of a dog model of radiation-induced injury after 3 months [41]. Ex-vivo evaluation of the irradiated field lesions consisted of perivascular fibrosis, mild myocyte degeneration, and mitochondria injury. Interestingly, no significant inflammatory cell infiltrate was detected, indicating that FDG accumulation was most likely related to microvascular damage and mitochondrial injury [41].

On the other hand, and despite the obvious technological advantages, the pre-clinical and clinical experience with quantitative myocardial perfusion PET imaging is limited to a small retrospective, cross-sectional study of 35 cancer survivors who underwent cardiac pharmacologic stress PET/CT at a median of 4.3 years (IQR 2.1–9.7) following thoracic irradiation [101]. The authors observed an inverse correlation between the mean cardiac radiation dose and global coronary flow reserve (CFR) and CFR in the LAD territory, even after adjustment for risk factors. Findings further support the possibility of coronary microvascular dysfunction as one of the potential underlying mechanisms of radiation-induced cardiotoxicity. However, this remains the subject of further investigation in future clinical trials. 

**Table 2:** Incidence and most common vascular distribution of radiation-induced myocardial perfusion imaging abnormalities.

Author; Year	N	Imaging Technique	Stress?	Months Post-RT	Incidence of PD	*p* Value vs. Pre-RT	RT Dose (Gy)	Defect Location
Hardenbergh, 2001 [96]	20	SPECT	No	6	60%	N/A	46–50	N/A
Lind, 2003 [97]	69	SPECT	No	6	N/A	<0.001	46–50	LAD
Lind, 2003 [97]	41	SPECT	No	12	N/A	0.07	46–50	LAD
Lind, 2003 [97]	16	SPECT	No	18	N/A	0.004	46–50	LAD
Marks, 2005 [98]	114	SPECT	No	6–24	32%	N/A	46–50	
Prosnitz, 2007 [102]	44	SPECT	No	36–72	68%	N/A	47.3	LAD
Chung, 2012 [103]	32	SPECT/CT	Yes	12	16%	0.68	50–52.2	N/A
Zellars, 2013 [104]	43	SPECT/CT	Yes	6	N/A	* 0.01	40–49	LAD

RT = radiation therapy; PD = perfusion deficit; LAD = left anterior descending coronary artery; SPECT = single photon emission computed tomography; CT = computed tomography. * *p*-value only significant for perfusion deficits at the apical segments during resting myocardial perfusion.

## 4. Immune Checkpoint Inhibitors-Related Toxicity

Immune checkpoint inhibitors (ICI) are monoclonal antibodies targeting the host immune negative regulation receptors, including cytotoxic T-lymphocyte–associated protein 4 (CTLA-4), programmed cell death receptor 1 (PD-1), and programmed cell death ligand 1 (PD-L1). These drugs can therefore modulate the immune system response and drive an immune-mediated attack against cancer cells with a demonstrated benefit in terms of overall prognosis and survival in a wide range of tumors [105]. However, the interaction with the immune system could also trigger a rare form of myocarditis, reported in approximately 0.1 to 1% of patients [106]. Despite being very rare, ICI-related myocarditis could be potentially lethal, with a mortality rate of up to 50%. This life-threatening complication is frequently observed during the first three months of treatment, particularly in patients undergoing combination therapy [107]. Although nonspecific ECG changes and elevated troponin are frequently observed, LVEF remains normal in about half the cases [106]. 

**Figure 5:**
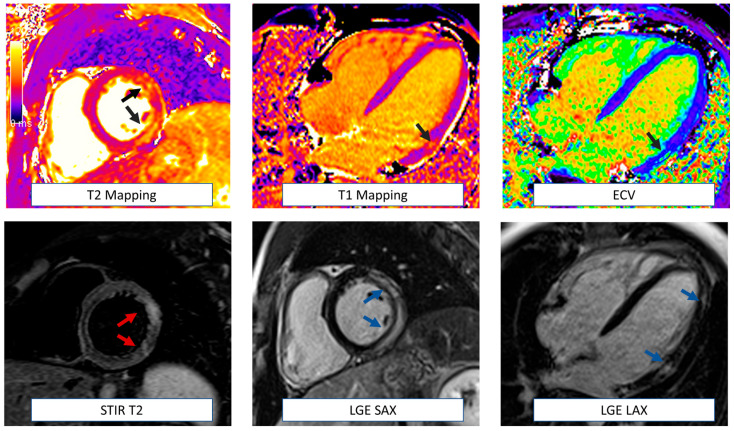
CMR in myocarditis. CMR is particularly helpful in diagnosing immune check point related myocarditis. The revised Lake Louise Criteria include at least one T2-based criterion (increase in regional or global T2 relaxation time or increased signal intensity in T2-weighted images) and one T1-based criterion (increased native T1 values, ECV or LGE). This patient showed increased signal intensity in short tau inversion recovery (STIR) T2 images (red arrows), increased T2 mapping values (black arrows), and increased T1 and ECV values in the lateral wall with mainly subepicardial LGE in the same region (blue arrows). ECV = extracellular volume. LGE = late gadolinium enhancement.

CMR is extremely helpful in the diagnosis of myocarditis [108]. The revised Lake Louise Criteria suggest the combined use of at least one T2-based (increased signal intensity in T2-weighted images or T2 mapping values) and one T1-based criterion (increased native T1 values, ECV or LGE) to increase the diagnostic accuracy [108] (Figure 5). Myocardial edema can be qualitatively detected as areas of increased signal intensity in STIR-T2 sequences [109]. This may be challenging in the case of global myocardial edema, where a semi-quantitative (a signal intensity ratio myocardium/skeletal muscle of ≥2.0) or quantitative (increased T2 mapping values) assessment should be necessary for diagnosis [109]. 

An international registry of 103 patients with ICI-associated myocarditis described LGE in approximately half of the patients, with predominant mid-wall (49%) and subepicardial (26.5%) distribution [110] and, more rarely, a diffuse or subendocardial pattern. The segments more frequently involved were the septum and the inferior and inferolateral wall, with a higher prevalence of LGE when the CMR was performed after the fourth day of admission [110] compared to within 4 days of admission. Increased signal intensity in T2-weighted short tau inversion recovery (STIR) images was found in 28% of patients. Only a subgroup underwent T1 mapping and ECV assessment with increased values compared to healthy individuals (1167.2 ± 32.9 ms and 34.3 ± 2.1%, respectively). Of note, 56 patients underwent cardiac biopsy; LGE was present in only 35% of positive histology, and an increased T2-weighted signal was present only in 26% of patients with lymphocytic infiltration. These findings are warranted as a reliance on LGE and T2 imaging approaches excludes ICI-induced myocarditis. Furthermore, nor the presence nor the pattern of LGE or an increased STIR-T2 signal were associated with MACE at follow-up. Instead, a reduced LVEF was associated with MACE (hazard ratio 2.07, 95% CI 1.10–3.93; *p* = 0.025) [110]. Increased native T1 mapping values demonstrated better association with myocardial injury when compared to T2 mapping values and excellent prognostic value in another study, being independently associated with subsequent MACE [111].

Molecular MRI imaging of myocarditis is being investigated in animal models and has not yet been translated into clinical practice. Maier et al. recently tested a new contrast agent targeting activated platelets consisting of microparticles of iron oxide (MPIO) conjugated to a single-chain antibody directed against ligand-induced binding sites (LIBS) of activated glycoprotein IIb/IIIa (=LIBS − MPIO). Histology confirmed CD41-positive cases, indicating platelet involvement in myocarditis in mice; quantification of the myocardial MRI signal confirmed a signal decrease after LIBS-MPIO injection [112]. This study demonstrated that platelets are activated in the inflammatory cascade in myocarditis and can be non-invasively imaged with LIBS-MPIO by molecular MRI in an animal model [112]. 

Detection of ICI-related myocarditis with radionuclide molecular imaging techniques is also possible and remains an area of ongoing investigation [113,114,115,116,117]. 

**Figure 6:**
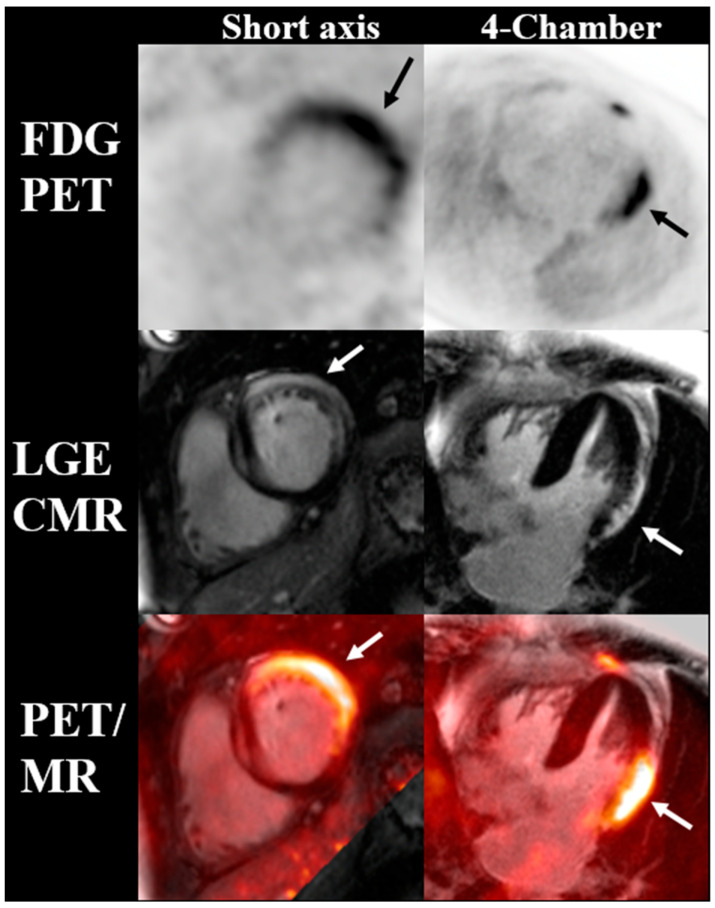
Cardiac FDG PET, CMR, and fused PET/MR images of a patient with suspected checkpoint inhibitor myocarditis. Myocardial FDG uptake matches the localization of late gadolinium enhancement along the subepicardial aspect of the lateral wall, indicative of active myocarditis (arrows).

Preliminary reports suggest that FDG-PET can be utilized to diagnose active ICI-related myocarditis, with one case report indicating that it can be helpful even in the absence of abnormalities on CMR [113,114]. When both imaging modalities are available, co-localization of FDG uptake on PET with T2 and/or LGE on CMR can further support the diagnosis of active myocarditis (Figure 6). This was shown in a prospective study of 65 patients who underwent simultaneous cardiac PET-MR for the evaluation of myocarditis, and the spatial agreement between FDG PET with T2 (kappa 0.75) and LGE (kappa 0.64) was substantial [115]. Compared with CMR (LGE and/or T2) as the reference, the authors estimated sensitivity and specificity for PET of 74% and 97% for the diagnosis of myocarditis, respectively [115]. However, the clinical experience with PET for the detection of acute or chronic (non-granulomatous) myocarditis remains limited, and consequently, its real diagnostic performance remains largely unknown. From a pathobiology perspective, FDG is a sensitive marker of cellular inflammation; thus, it is conceivable that FDG PET may be as sensitive as CMR for the detection of myocarditis. The main issue with FDG PET is the need for dietary preparation to induce a “metabolic switch” to suppress physiologic myocardial glucose uptake, an inherent limitation which affects or reduces the specificity of the test to differentiate between nonspecific and pathologic uptake. This metabolic switch can be induced by strategies that increase fatty acid and/or ketones levels and, at the same time, reduce insulin release, including prolonged fasting and a dietary switch to a lipid-rich/carbohydrate-deprived (a.k.a. ketogenic) diet prior to the exam [116]. Retrospective data from convenient cohorts suggest that myocardial FDG suppression is achieved in 81–84% of subjects following the ketogenic diet for at least 24 h [117,118], but higher suppression rates (up to 95%) have been recently shown with longer periods of ketosis (e.g., 72 h) [119,120]. Emergent data also suggest that beta-hydroxybutyrate levels, a marker of ketosis, may assist in the identification of individuals who failed to make this metabolic switch [121,122]. On the contrary, supplementary strategies intended to further raise fatty acid and/or ketone levels, including a fatty load [117] and ketone ester drink [121], prior to FDG injection, have actually shown discouraging results to suppress FDG uptake in the heart in well-conducted randomized clinical trials.

However, the need for a dietary preparation to suppress physiologic myocardial glucose uptake is an inherent limitation, which affects or reduces the specificity of the test to differentiate between nonspecific and pathologic uptake. 

Imaging of the somatostatin receptor using 68Ga-DOTATATE and ^68^Ga-DOTATOC has also been studied in patients with suspected ICI myocarditis [123,124]. These scans do not require dietary preparation, and one study suggested that ^68^Ga-DOTATOC uptake may precede LGE and T2 elevation on CMR. Furthermore, one study found that myocardial ^68^Ga-FAPI uptake was higher in patients with suspected ICI myocarditis compared to patients receiving ICI without evidence of cardiac involvement, suggesting that ^68^Ga-FAPI should be studied more extensively in this patient population [113,125].

**Figure 7:**
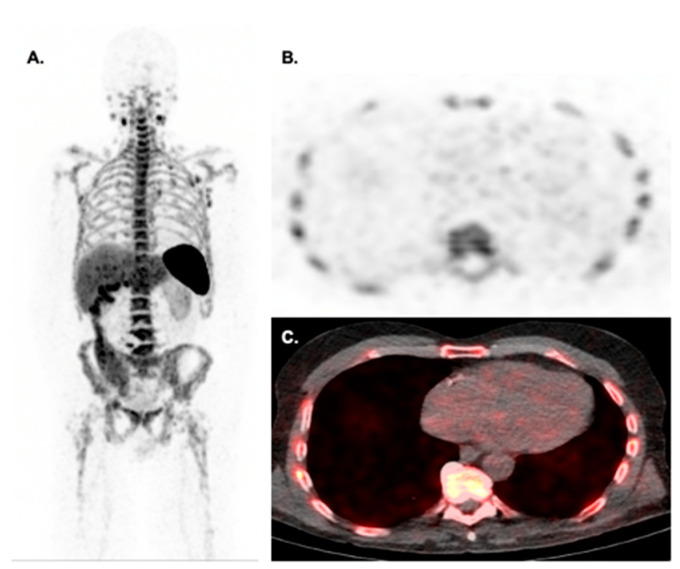
Biodistribution of CD8 PET imaging with 89Zr-Df-IAB22M2C in a 71-year-old male with metastatic melanoma on pembrolizumab for 28 days at the time of imaging. Maximal intensity projection images (**A**) show multinodal CD8+ involvement in the neck, axilla, and mediastinum. Please notice the low level of myocardial uptake on axial PET (**B**) and fused PET/CT (**C**) images make this radiotracer suitable for detecting checkpoint inhibitor myocarditis. Images courtesy of Dr. Michael Farwell from the University of Pennsylvania.

However, the future of molecular imaging is geared towards the development of more disease-specific or targeted probes. In this sense, there is a significant interest in developing and testing novel immunotherapeutic drugs, including PD-1 and PD-L1 PET probes (thus imaging the targets of ICI) [48,53], radiopharmaceuticals targeting CD8+ T cells [126], and T-cell activation markers (e.g., CD69) [127]. Most of these probes are currently in the pre-clinical stage, and their cardiovascular translation remains uncertain, although it is conceivable that imaging cardiomyocyte expression of PD1 and PD-L1, or imaging activated CD8 cells in the heart, may lead to the identification of ICI myocarditis (Figure 7). 

## 5. Challenges and Future Perspectives

As anti-cancer treatments continue to evolve and survival from cancer improves, the role of a multidisciplinary cardio-oncology team becomes of utmost importance in the management of these complex patients through a more personalized approach, from surveillance and early identification of cardiotoxicity to prevention and therapeutic management of complications. Cardiovascular imaging has evolved as a key tool to this purpose, allowing a non-invasive evaluation complementary to biomarkers and clinical assessment. In this regard, advanced imaging techniques offer the advantage of tissue characterization that, beyond LV function assessment, can provide early identification of cardiac injury with possible impact on therapeutic management and prognosis. Although reliable, accurate, and reproducible, a routinary use of these advanced techniques may be limited in clinical practice by high costs, long acquisition time, and availability of the modality. The application of rapid CMR protocols that are proven to provide high diagnostic accuracy in an average scan time of approximately 15 min (12 ± 4 for a non-contrast study) may increase the overall availability of the modality [128]. The most recent implementation of machine learning for LVEF assessment, capable of significantly reducing the image analysis time (186× faster than human), is another promising strategy for this purpose [129]. Finally, novel CMR techniques capable of non-invasively providing information regarding cardiac metabolism and energetics, although currently mainly used only in pre-clinical and research contexts, may further change the clinical scenario in the future. Radionuclear and molecular imaging enable precise examination of the underlying pathophysiology and, thus, represent a unique component for personalized medicine in cardio-oncology by facilitating detection and surveillance. A novel set of radiotracers capable of detecting activated fibroblasts, macrophages, and ROS production have great potential to further unravel cardiotoxicity mechanisms in the future. 

## Figures and Tables

**Table 1:** Summary of radiotracers, mechanisms of uptake, and targets for imaging cardiovascular inflammation, injury, and healing response in cancer-related cardiotoxicity.

Tracer	Mechanism of Uptake	Cellular Target (s)	Application
^18^F-FDG *	Glucose transporters 1 and 3	Activated granulocytes ∫ and mononuclear cells ∮, ischemic/hypoxic myocytes	Inflammation/injury
^68^Ga-Dotatate */Dotatoc	Somatostatin receptor sub-type 2	Activated mononuclear cells ∮	Inflammation
^18^F-FLT	Thymidine kinase 1	Proliferating granulocytes ∫ and mononuclear cells ∮	Cellular proliferation
^18^F-FMISO	Nitroreductase enzymes	Hypoxic cardiomyocytes	Hypoxia
^18^F-DHMT **	Reactive oxygen species	Activated macrophages, cardiomyocytes under oxidative stress	Cytotoxicity
^18^F-NOS	Nitric oxide synthase	Activated macrophages, cardiomyocytes under oxidative stress	Inflammation
^68^Ga-pentixafor	CXCR4	Active monocytes and macrophages, cardiomyocytes under stress	Inflammation
^68^Ga-DOTA-ECL1i **	CCR2	Activated monocytes and macrophages	Inflammation
Cu-ATSM	Mitochondrial Electron Transport System	Hypoxic cardiomyocytes	Hypoxia
^68^Ga-FAPI	Fibroblast activation protein inhibitor	Activated fibroblasts	Fibrosis
^123^I-MIBG	Norepinephrine transporter uptake	Pre-synaptic neurons	Sympathetic innervation
^111^In-antimyosin	Exposed myosin	Sarcolemmal damage	Necrosis
^123^I-BMIPP	α-oxidation and β-oxidation	Metabolically active cells	Fatty acid metabolism

* FDA-approved radiotracer for clinical use; ** pre-clinical phase. No published human data on cardiotoxicity. ∫ granulocyte cells = neutrophils, eosinophils, basophils, and mast cells. ∮ mononuclear cells = lymphocytes, monocytes, and macrophages. FDG = fluorodeoxyglucose; FLT = fluorothymidine. MMP = matrix metalloproteinases; BMIPP = beta-methyl-p-iodophenylpentadecanoic acid; MIBG = metaiodobenzylguanidine; HER2 = human epidermal growth factor receptor 2.

## Data Availability

Not applicable.
